# Guest Editorial Special Section on Functional Recovery and Brain Plasticity

**DOI:** 10.1109/OJEMB.2023.3339954

**Published:** 2024-01-05

**Authors:** Alessandra Pedrocchiguest, Eleonora Guanziroli

**Affiliations:** NEARLAB, Neuroengineering and Medical Robotics Laboratory, AND WE-COBOT, Wearable Collaborative Laboratory, Department of Electronics, Information and BioengineeringPolitecnico di Milano 18981 Milan Italy; Villa Beretta Rehabilitation CenterValduce Hospital 220196 23845 Costa Masnaga Italy

## Abstract

The aim of rehabilitation after neurological damage is functional recovery, which includes motor, sensory, and cognitive aspects, which are closely interrelated [22].

The aim of rehabilitation after neurological damage is functional recovery, which includes motor, sensory, and cognitive aspects, which are closely interrelated [22].

When successful recovery from brain damage is achieved, what happens is that the organization of brain connections is modulated to compensate for the parts of the brain that have been damaged and are therefore no longer functional [10].

To achieve this recovery the brain has redundancy and plasticity [17]. Thanks to redundancy, the same function is mapped and controlled by a system of neuronal structures that work together and in parallel. Plasticity is the ability of the nervous system to change its activity in response to intrinsic or extrinsic stimuli by reorganizing its structure, functions, or connections [27]. Together, these properties lead to changes in the structure and connectivity of networks in the brain over time, resulting in relearning and thus functional recovery [24]. Brain plasticity can be modulated by rehabilitative intervention after an acute event or during the phases of the disease [29].

This opens a window to define exercise as a beneficial drug that can be prescribed as a medicine to promote biological, neurobiological and epigenetic changes, exploiting brain plasticity [15]. The response of neurons is, indeed, dominated by a limited subset of specific inputs that represent a small minority of the total input repertoire anatomically projecting to that brain area [8]. The brain can change the strength of the connections allowing formerly “ineffective” inputs to dominate this zone [14].

Changes are by nature limited in size and achieved by repetition, behaviorally important inputs will be strengthened, and noncontributing inputs will be weakened. Neurotransmiters regulate these changes, e.g., Acetylcholine, Noradrenaline are released when brain is engaged with unexpected stimuli and attention is focused, while Dopamine is released upon successful target achievement [30].

What levers are available for designing rehabilitation interventions to facilitate this relearning process?

Firstly, we need to recognize that the process we are analyzing leverages a closed-loop system consisting of the interaction of three main elements: the sensory system, the neural control and the musculoskeletal system.

All three actors are fully involved in the process of relearning and functional recovery through a process of co-adaptation. The more rehabilitation activates the three actors in a synergistic and coordinated way, the more the relearning mechanisms can be facilitated [31].

What is the role of technology in this process?

The first answer is that the role of technology is not unique, but multiple and diverse.

According to the principles of neuroplasticity and motor learning, hi-tech devices have been introduced in the rehabilitation process since they can maximize afferent input from peripheral joints and provide task-specific stimulation to the central nervous system to promote neuroplasticity.

Increasing therapy dosage, intensity, number of repetitions, execution of task-oriented exercises, and combing top-down and bottom-up approaches, can promote plasticity and functional recovery [33].

Historically, we have seen technologies entering the rehabilitation at increasing complexity. Digital exercises, called exergames, are mostly used app integrated with screens and tablets. In this context, the technologies flank the rehabilitation exercises traditionally proposed by the physiotherapist and occupational therapist, for example through augmented reality exercises and exergames, enriching the rehabilitation pathway with greater training intensity, enriching the complexity of stimulation, increasing motivational and, opening also the opportunities for remote monitoring and home-based treatments [25].

To increase the level of complexity and reach patients with greater deficits, robotic exercises, first manipulandum's and then exoskeletons, have entered clinical practice, adding to the previous elements, also support for the execution of ecological motor tasks. This way, the subject experience has been enriched not only by motor support but also by enrichment of the associated sensory proprioceptive information [12].

More recently, the design focus in the field of rehabilitation robotics has increasingly been on the integration of the patient's residual capacity in the execution of the assisted movement, to ensure maximum patient engagement in the exercise. Impedance controller with direct torque measurement can be used to provide positive-feedback compensation useful to promote good transparency and render both compliant and stiff behavior at the joint actuated [11].

Another critical point that characterizes the entry of technology into the rehabilitation clinical context concerns neuromodulation. By neuromodulation we mean, in the broadest sense, all systems that interface with the nervous system and stimulate it to modulate its function [3].

This definition includes training with Functional Electrical Stimulation (FES), which stimulates the peripheral nerve using surface electrodes to reproduce functional muscle contraction and therefore providing the tasks execution with the associated spindle feedback. FES has large use in daily clinics, in simple motor tasks, to improve muscular strength at single joint, or during the execution of multi-joints tasks where inter-limbs, inter-joints coordination is required. A well-established task is FES cycling [16], [2]. More at research level, we have seen FES-mediated upper limb training [5] and gait training [26].

The FES correlates of brain plasticity are still to be fully understood even though some studies with animals [38] and with humans by fMRI [18], [19] and studying reflexes modulation [32] are shedding some lights on this.

Neuromodulation can be used to provide sensory stimulation, peripheral sensory stimulation, such as Transcutaneous Electrical Nerve Stimulator (TENS) to manage pain and provide proprioceptive feedback [7] and further, in sensorial neuroprosthetics using implanted neural interfaces, which are now more commonly used in amputees than in rehabilitation [35] [37].

Finally, neuromodulation has been targeted to the central nervous system.

Multiple technological solutions have been proposed for brain stimulation solutions, from Transcranical Direct Current Stimulation (TDCS) [6], [13] to Transcranial Magnetic Stimulation (TMS) [21], [28] to transcutaneous auricular Vagus Nerve Stimulation [39]. Despite the high impact of these research in the neuroscientific community, from the clinical point of view, these solutions are still far from daily clinical practice and are still considered as research tools and prototypes.

Lastly, but not less relevant, Spinal Cord Stimulation has been largely proposed in the latest years, mostly as implanted solution after Spinal Cord Injury to restore motor and autonomic functions [4], [34], [36], but more recently also for rehabilitation purposes with trancutaneuous electrodes [9], [23].

In this Special Issue, we have collected contributions of very relevant research on these aspects, with the aim of describing the technology and documenting its clinical efficacy, but also, and above all, to propose its neurophysiological interpretation, i.e., the effect in term of brain plasticity and remodeling.

In this context, we have in fact a work [A1] on digital technologies based on tablets, to validate the use of a new app called QDraw. This digital app provides a quantitative analysis of drawings and allows to investigate whether the results of this analysis can reveal distortions of body representations in chronic stroke patients.

We have one paper on exoskeletons, studying the effect on cognitive load and neurotrophic factors [A2] in the brain associated to exoskeletons training.

Thirdly, on neuromodulation applications, we have a very interesting paper [A3] on transcutaneous auricular Vagus Nerve Stimulation in the rehabilitation clinic in which cognitive and motor changes induced by stimulation are evaluated.

Still about neuromodulation, we also included a paper [A4] analysing the state of the art in the use of ultrasound stimulation in the treatment of stroke.


Alessandra Pedrocchi, Guest Editor
NEARLAB, Neuroengineering and Medical Robotics Laboratory
WE-COBOT, Wearable Collaborative Laboratory
Department of Electronics, Information and Bioengineering
Politecnico di Milano 20133 Milano, Italy
e-mail: alessandra.pedrocchi@polimi.it
Eleonora Guanziroli, Guest Editor
Villa Beretta Rehabilitation Center
Valduce Hospital
23845 Costa Masnaga (LC), Italy
e-mail: eleonora.guanziroli@gmail.com


## Appendix Related Articles

[A1] I. Martinelli et al., “A quantitative, digital method to analyze human figure drawings as a tool to assess body representations distortions in stroke patients,” *IEEE Open J. Eng. Med. Biol.,* vol. 4, 2023, doi: 10.1109/OJEMB.2023.3277711.
[A2] A. V. R. Cataldo et al., “Enhancing neuroplasticity in the chronic phase after stroke: Effects of a soft robotic exosuit on training intensity and brain-derived neurotrophic factor,” *IEEE Open J. Eng. Med. Biol.,* vol. 4, 2023, doi: 10.1109/OJEMB.2023.3313396.
[A3] M. Colombo, S. Aggujaro, N. Lombardi, A. Pedrocchi, F. Molteni, and E. Guanziroli “Motor and cognitive modulation of a single session of transcutaneous auricular Vagus Nerve Stimulation in post stroke patients: A pilot study,” *IEEE Open J. Eng. Med. Biol.,* vol. 4, 2023, doi: 10.1109/OJEMB.2023.3268011.
[A4] M. M. Yüksel et al., “Low-intensity focused ultrasound neuromodulation for stroke recovery: A novel deep brain stimulation approach for neurorehabilitation?,” *IEEE Open J. Eng. Med. Biol.,* vol. 4, 2023, doi: 10.1109/OJEMB.2023.3263690.
